# A Clustering Framework for Monitoring Circadian Rhythm in Structural Dynamics in Plants From Terrestrial Laser Scanning Time Series

**DOI:** 10.3389/fpls.2019.00486

**Published:** 2019-04-17

**Authors:** Eetu Puttonen, Matti Lehtomäki, Paula Litkey, Roope Näsi, Ziyi Feng, Xinlian Liang, Samantha Wittke, Miloš Pandžić, Teemu Hakala, Mika Karjalainen, Norbert Pfeifer

**Affiliations:** ^1^Department of Remote Sensing and Photogrammetry, Finnish Geospatial Research Institute, National Land Survey of Finland, Helsinki, Finland; ^2^Department of Remote Sensing and Photogrammetry, Centre of Excellence in Laser Scanning Research, National Land Survey of Finland, Helsinki, Finland; ^3^Department of Built Environment, Aalto University, Espoo, Finland; ^4^University of Novi Sad, BioSense Institute, Novi Sad, Serbia; ^5^Department of Geodesy and Geoinformation, Technische Universität Wien, Vienna, Austria

**Keywords:** laser scanning, time series, structural dynamics, circadian rhythm, phenology

## Abstract

Terrestrial Laser Scanning (TLS) can be used to monitor plant dynamics with a frequency of several times per hour and with sub-centimeter accuracy, regardless of external lighting conditions. TLS point cloud time series measured at short intervals produce large quantities of data requiring fast processing techniques. These must be robust to the noise inherent in point clouds. This study presents a general framework for monitoring circadian rhythm in plant movements from TLS time series. Framework performance was evaluated using TLS time series collected from two Norway maples (*Acer platanoides*) and a control target, a lamppost. The results showed that the processing framework presented can capture a plant's circadian rhythm in crown and branches down to a spatial resolution of 1 cm. The largest movements in both Norway maples were observed before sunrise and at their crowns' outer edges. The individual cluster movements were up to 0.17 m (99th percentile) for the taller Norway maple and up to 0.11 m (99th percentile) for the smaller tree from their initial positions before sunset.

## Introduction

Terrestrial Laser Scanning (TLS) measures its surrounding 3D environment using a dense point cloud. It has become a staple in several research fields, including forest sciences (Dassot et al., 2011; Liang et al., 2016), landslide monitoring (Jaboyedoff et al., 2012), building change detection and deformation monitoring (Mukupa et al., 2017), and glaciology (Deems et al., 2013). The strength of TLS lies in its rapid data collection, regardless of external lighting conditions. Objects dozens of meters from the scanner can be mapped with sub-centimeter-level spatial resolution in minutes. This enables fast and accurate digitization of static scenes, both day and night. Compared with other terrestrial point cloud sources (e.g., image-based and personal laser scanning), TLS has the highest digitization accuracy and a unique capability of delineating crown structure (Liang et al., 2015).

In addition to collecting standard forestry parameters, dense TLS point clouds allow accurate object modeling in forests. The previous literature lists several different methods involving point cloud voxelization and skeletonization (Bucksch and Lindenbergh, 2008; Bucksch et al., 2010; Livny et al., 2010; Schilling et al., 2012; Bremer et al., 2013; Eysn et al., 2013). More recently, various quantitative structural (e.g., Raumonen et al., 2013; Delagrange et al., 2014; Hackenberg et al., 2015) and stem curve modeling techniques (e.g., Kelbe et al., 2013; Yu et al., 2013; Liang et al., 2014; Wang et al., 2016) have gained popularity in determining forest parameters for both forest management and ecology applications (Calders et al., 2014; Jaakkola et al., 2017). However, to our knowledge no techniques have been applied for short interval here, <1 h TLS time series for monitoring the temporal development of individual tree segments.

Although TLS is used routinely in many research applications, its utilization has thus far been limited to studying circadian rhythms in plant physiology. In a recent review Eitel et al. (2016) divide LiDAR applications by temporal resolution into either “multi-temporal” or “hypertemporal” applications. The former entails scanning repetition over 1 month intervals; the latter entails intervals of with <1 month. This definition is suitable for longer term phenomena such as inter- (Liang et al., 2012; Culvenor et al., 2014) and intra (Portillo-Quintero et al., 2014; Calders et al., 2015) seasonal phenology, annual biomass change (Srinivasan et al., 2014; Crommelinck and Höfle, 2016), and growth dynamics (Griebel et al., 2017). However, only a few studies using commercially available TLS systems have demonstrated that the technique is feasible for detecting physiological plant phenomena at timescales shorter by one order of magnitude or more (Puttonen et al., 2015, 2016; Zlinszky et al., 2017; Herrero-Huerta et al., 2018). Similar short timescale measurements using TLS for monitoring lava flows (Crown et al., 2013) and structural deformations (Grosse-Schwiep et al., 2013) have been demonstrated, however.

The capability of mapping plant' short-term structural dynamics would afford valuable means for plant sciences to test open hypotheses concerning circadian motion and foliar nyctinasty. In his review, Minorsky (2019) lists several hypotheses concerning foliar nyctinasty that have been presented over the last century. These include internal plant processes such as better temperature control and water shedding. He also discusses more novel bithropic (plant-herbivore) and tritrophic (plant-herbivore-predator) interaction hypotheses, whose testing would require accurate information on the plant canopy structure at different times. Light Detection and Ranging (LiDAR)—TLS included—data collection offers strong potential as a solution. LiDAR data collection does not interfere with internal plant processes. LiDAR measurements also provide directly quantified digitized information on several plants' structure in a single measurement which may be even dozens of meters tall. These properties open entirely new opportunities for monitoring internal tree processes, creating new dynamic structural models of canopy scale, and studying plants' interaction with their surroundings in natural growth conditions. Full utilization of this LiDAR data potential requires easily implemented robust workflows with well-documented performance metrics. This study's goal was to develop a workflow for monitoring circadian rhythm dynamics—such as cyclical drooping of leaves and branches—in plants with a temporal point cloud data series collected using multiple static terrestrial laser scanners. To our knowledge, the 3D point cloud workflow the study presents is among the first to capture the circadian rhythm in plant dynamics in outdoor conditions of this scale. Workflow is based on spatial point cloud clustering and monitoring cluster movement over time. This allows the user to monitor each point cluster. Alternatively, targets' overall movement patterns may be captured by aggregating cluster information.

This study's method was based on reviewing earlier research approaches utilizing point cloud data collected using LiDAR or calculated from overlapping imagery. Table 1 lists some more commonly cited examples of earlier studies, and their strengths and weaknesses in structural plant dynamics monitoring. When point cloud data collection techniques are considered, LiDAR- and imaging-based approaches exhibit complementary properties: LiDAR performs better in varying and unlit lighting conditions, and penetrate plant canopies better. On the other hand, imaging methods provide color information and can capture data for limited plant parts more quickly, which is important during daylight with more airflows.

**Table 1:** Methodological review of possible methods to model structural plant dynamics in outdoor conditions.

	**Common**		**Method specific**			**Example use cases**	**References**
**Technique**	**Strengths**	**Weaknesses**	**Name**	**Strengths**	**Weaknesses**		
Laser scanning	Direct 3D information acquisition; Data acquisitions not limited by external lighting conditions	Data acquisition times—sensitivity to wind; Internal occlusions in the object; Single wavelength only	*Presented framework*	*Straight forward processing; Limited parameter number; Cluster movement tracking*	*Does not separate individual plant parts; No direct cluster shape detection*	Circadian rhythm monitoring of plants over wide area	–
			Height percentiles	Fast to perform; No parametrization	Cannot track specific plant part movements; Overgeneralizes the movement patterns	Circadian rhythm monitoring of plants over wide area	Puttonen et al., 2016; Zlinszky et al., 2017
			Quantitative structure modeling (QSM)	Robust branch and stem estimations; Plant part volume and length estimations; Plant part movement tracking	Work best in leaf-off conditions; Robustness to the internal occlusions; Computationally heavy	Accurate estimates for tree stem and branch length, diameter, and volume in forestry and ecological applications	Raumonen et al., 2013; Hackenberg et al., 2015
			Skeleton modeling	Robust branch and stem length and angle estimations; Plant part movement tracking	Work best in leaf-off conditions; Robustness to the internal occlusions; No volume information	Localized plant point cloud registrations between separate data acquisitions; Phenological trait estimation in plants and trees	Bucksch and Khoshelham, 2013; Wu et al., 2019
Imaging	Individual image acquisition nearly instantaneous; Multiple wavelength bands	Sensitivity to external lighting conditions; Range information not directly available (planar geometry); Weak penetration through the canopy surface	Structure from motion	High density 3D surface models; Individual plant part monitoring	Wide area coverage difficult (high overlap required); Computationally heavy	Phenotype parameter reconstruction; Leaf parameter estimation	Li et al., 2013; Duan et al., 2016; Hui et al., 2018
			RGB	Affordable instrumentation	Wavelength band number	Time lapse generation of circadian movements	Gooch et al., 2004
			Multi- and hyperspectral	Plant part differentiation performance	Lower resolution; Slower acquisition	Plant health estimates; Detection of active sites	Pan et al., 2015
			Thermal	Can measure in dark; Can show plant processes not visible and near-infrared wavelengths	Low resolution	Heliotropism monitoring in sunflowers	Atamian et al., 2016

*The method review is not exhaustive*.

In general, the modeling methods seek either an accurate structural description—like QSM (Raumonen et al., 2013; Hackenberg et al., 2015)—or generalize the movement using point cloud statistics (e.g., Puttonen et al., 2016; Zlinszky et al., 2017). The former methods are computationally heavy and prone to structural changes due to internal canopy movements and occlusions. The generalizing methods have lower spatial resolution and cannot capture all movement patterns due to overgeneralization. Here, the proposed method seeks to find a compromise between high accuracy models and overgeneralization by creating initially small cluster sizes while not fixing their exact shape and size over time.

The method development's main assumption was that the changes in the object point cloud were only due to the object's intrinsic movements: The measurement scene was assumed to be stable. Factors affecting the measurement scene stability were therefore monitored during data collection and filtered out before data processing. The following factors affecting measurement stability were recognized:

- Random external factors, such as wind, rain, or other actors (e.g., animals), disturbing the assumption of slow and systematic object movement.
- Abrupt or significant spatial changes, such as a snapping tree branch, causing target deformation, and breaking the systematic internal target dynamics.
- Significant changes in the object's visibility, i.e., being partly or fully occluded from all scanners due to occlusions resulting from external objects or from internal self-occlusion (e.g., a branch drooping and occluding other branches behind it).

## Materials and Methodology

### Test Site

**Figure 1:**
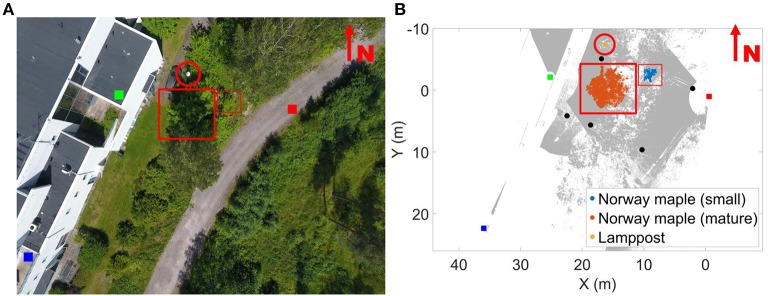
Overview of the measurement area **(A)** and the top view of its point cloud **(B)**. Scanner locations are marked with colored boxes: being the FARO Focus X330 (red); FARO Focus 3D 120 S 1 (dark blue); and TRIMBLE T5X (green). The gray points denote the combined point cloud of all three scanners. All targets monitored in the study are marked individually in the figure and highlighted with a red circle (lamppost) and rectangles (Norway maples). The five reference sphere locations are marked with black disks on the point cloud. The overview image on the left was taken at a different time than the measurement and is added for information. It is not to the same scale as the point cloud figure.

Measurements were taken in southern Finland (Kirkkonummi, N. 60° 9.674′, W. 24° 32.807′) on August 24 and 25, 2016 in leaf-on conditions. The measurement site was on a shallow southeast facing slope. The site was scanned with three terrestrial laser scanners mounted on tripods (Figure 1). FARO Focus 3D X 330 was located on the road next to the FGI building. The two other scanners were located on the roofs of the building (FARO Focus 3D S 120—southern, Trimble TX5—northern). The height difference between the scanner on the road and those on the roofs was about 10 m. The test site, about 35 m x 36 m, included several fully grown trees, understory, and operator-placed objects, including five reference spheres of 0.099 m radius. The experiment focused on a fully grown *large* and a *small* Norway maple (*Acer platanoides*). To test the internal point cloud dynamics in objects, a static object was also monitored: a lamppost next to the FGI building (Table 2).

**Table 2:** Targets measured during the experiment.

**Target name**	**Latin name**	**Bounding box dimensions (m)**			**Point number average and standard deviation**
		***x***	***y***	***z***	
Norway maple (large)	*Acer platanoides*	7.05	6.92	9.10	3,191,100 ± 30,500
Norway maple (small)	*Acer platanoides*	2.19	2.32	6.19	231,200 ± 2,500
Lamppost	*Columna lucerna*	0.95	0.76	4.40	45,300 ± 300

*The target bounding boxes are calculated from the points of the initial scans measured at 20:10 h. Point number averages and their standard deviations are calculated from all DAIs used in the analysis. Object point clouds were delineated manually from the combined point cloud of all three scanning locations. A buffer zone was left around each object volume to accommodate any systematic movements in the object point cloud during the experiment. Reported bounding box dimensions, point number averages and standard deviations were calculated after all pre-processing steps*.

The test site was scanned repeatedly from sunset to sunrise. Measurements were takes for a total of ~14.5 h, covering twilight and night (about 9 h in total). A total of 130 separate scans was collected, with the three stationary laser scanning systems placed around the site. One hundred and twenty-three of 130 scans representing 41 Data Acquisition Intervals (DAI) were selected when the airflows had settled. Each scanner used a predefined scanning parameter set during data collection. Scan intervals were about 20 min, during which operators started the scanners individually. Scanning times for all scanners were comparable, at about 12 min the preset scanning parameters.

Weather conditions were stable during data acquisition. The air was calm during the night, with no wind or occasional gusts (qualitative observation). There was no rainfall during measurements. Possible water condensation on leaves on lower branches was verified during the night. The scanner operators recorded current measurement conditions, including measurement time (t), temperature (T), atmospheric pressure (p), relative humidity (RH%), relative cloudiness (qualitative observation), and wind (qualitative observation) in logbooks. The entire measurement was also documented with a time lapse video created from images taken with a 1 min interval using a GoPro4 Black (GoPro Inc., San Mateo, CA, USA) camera (Supplementary Video 1).

### Measurement Systems

All three laser scanner systems were phase-based (Table 3).

**Table 3:** Property comparison of the terrestrial laser scanning systems used in measurements.

**Scanner**	**FARO Focus X330**	**FARO Focus 3D 120 S**	**TRIMBLE T5X**
Type	Continuous	Continuous	Continuous
Wavelength (nm)	1,550	905	905
Laser class	3R	1A	1A
Scanning mechanism	Vertically rotating mirror, horizontally rotating base	Vertically rotating mirror, horizontally rotating base	Vertically rotating mirror, horizontally rotating base
Maximal FOV	360/300	360/305	360/300
Maximal scan frequency	976,000	976,000	976,000
Range (m) (90% reflectance)	330	120	120
Distance accuracy (25 m)	0.3 mm, 90%; 0.5 mm, 10%	0.95 mm, 90%; 2.20 mm, 10%	0.95 mm, 90%; 2.20 mm, 10%
Beam divergence (rad)	0.19	0.19	0.19
Beam diameter at exit	3.0 mm	3.0 mm	3.0 mm

**Figure 2:**
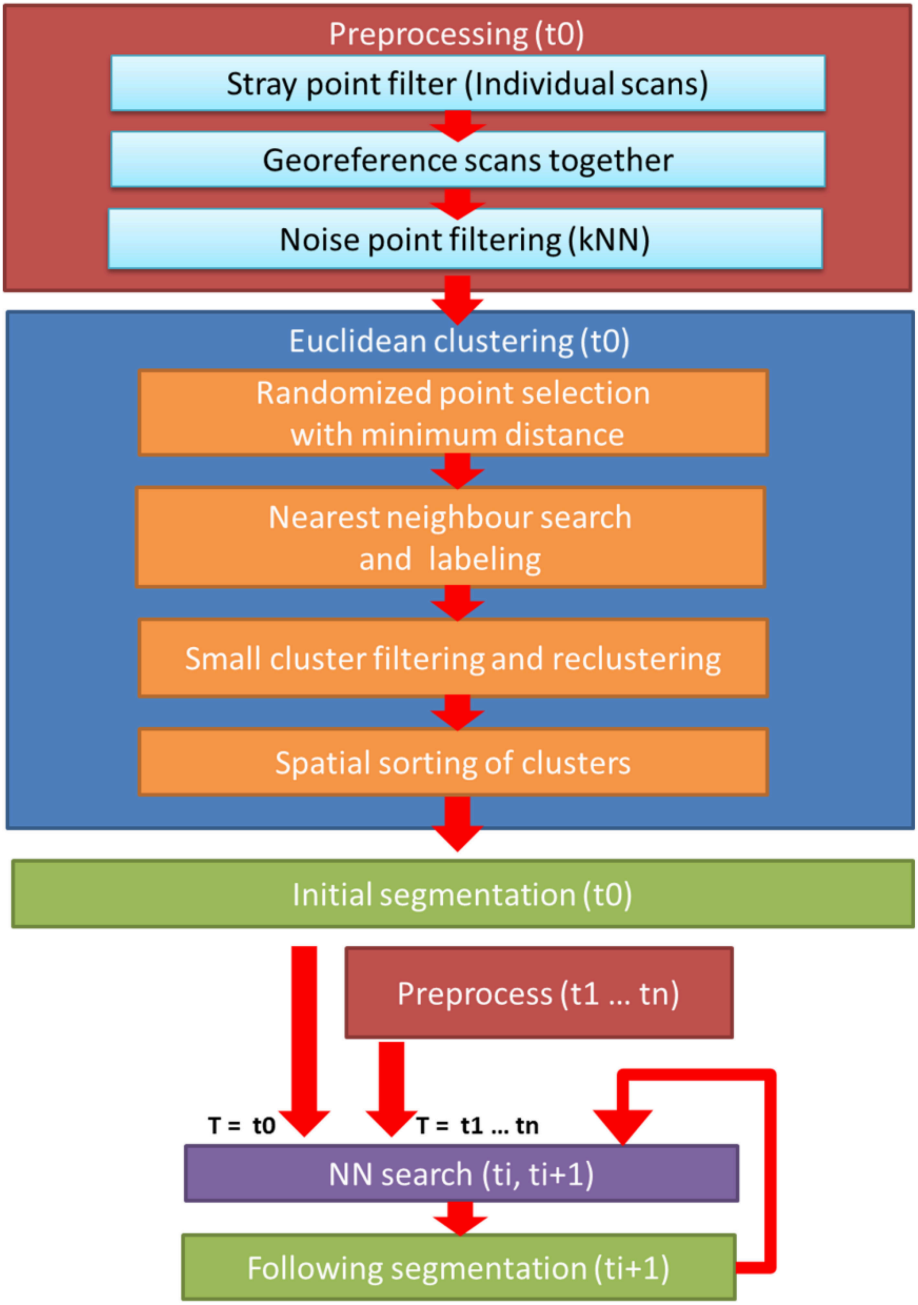
Flowchart of the cluster monitoring process. Workflow consists of three main steps: for each DAI, pre-processing of the merged point cloud of three scanners (red); initial clustering for the object point cloud at the first DAI (t0, blue); and the iterative nearest neighbor clustering cycle for the point clouds in all remaining DAIs (violet).

The temporal data collected was divided into DAIs. Point cloud processing consisted of two main steps, preprocessing, and clustering. These steps were performed similarly to the point clouds of each DAI, except for the initial DAI where a different clustering approach was used. The point cloud processing workflow is presented in Figure 2.

### Point Cloud Preprocessing

First, the data were pre-processed with FARO SCENE software (v5.4). Second, the five reference spheres were selected and labeled for each scan manually. A sphere with a radius of 9.9 cm was then fitted on each. Third, all point clouds were co-registered using sphere locations and other sensor data including scanner inclinometer, altimeter, compass, and GPS. After co-registration point clouds were filtered using FARO's stray point filter (3 × 3 grid, 0.02 m distance threshold and 50% allocation threshold). Points with raw intensity value <650 were also removed.

The pre-processed point clouds were exported into compressed.laz format using lastools software (https://rapidlasso.com/). The two study objects and all reference spheres were then manually segmented and labeled in each DAI using the boundaries defined for the first DAI. In labeling each object was delineated several times using 2D projections from different viewing angles. The MATLAB tools used in delineating target point clouds are available at ResearchGate (https://www.researchgate.net/publication/316990245_Point_cloud_cutting_scripts_for_MATLAB). The initial delineation boundaries were then used for all subsequent DAIs to extract the targets from the whole point cloud. A buffer zone was left around each object boundary in the initial delineation to accommodate any systematic movements in the object point cloud during the experiment. In the remaining text a delineated point cloud of one object is called “an object point cloud.”

### Object Point Cloud Clustering Over Time

In this phase each delineated object point cloud in each DAI was clustered. The clustering workflow started with a two-phase initialization, which was performed only for the first DAI. In the following DAIs, an iterative nearest neighbor clustering was performed. The workflow flowchart is presented in Figure 2. All clustering and cluster monitoring steps in the study were conducted with MATLAB 2017a (Mathworks Inc., Natick, MA, USA).

In every DAI, the pre-processed object point clouds were filtered to remove isolated points. The filter rule was to remove points that with fewer two neighbors in a range of 0.01 m.

**Figure 3:**
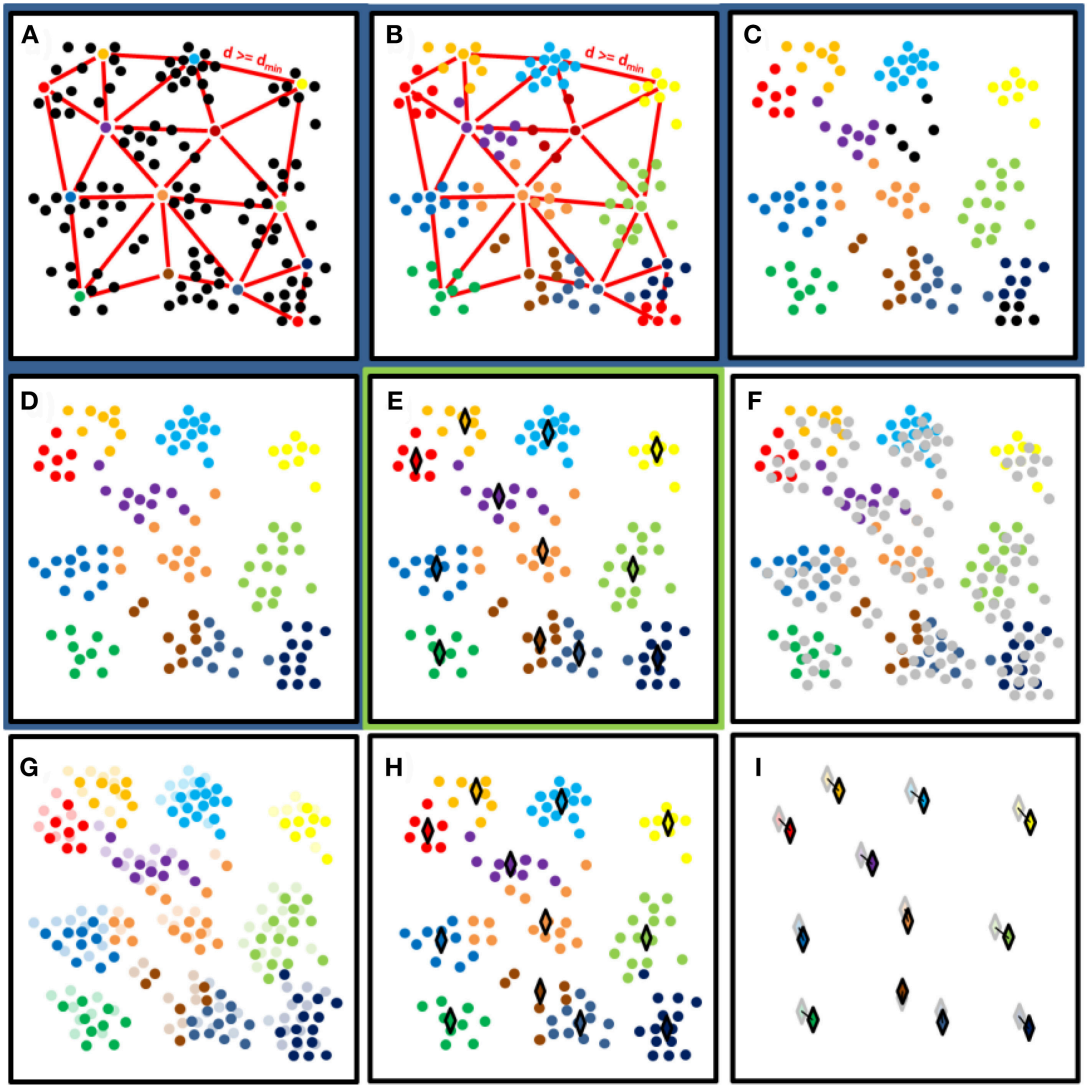
A synthetic example of the clustering process presenting processing steps in panels. Panels with the dark blue background **(A–D)** show the initial cluster determination process for the first DAI. Panel **(E)** with light green background shows the result of the initial clustering. Panels **(F–I)** show the nearest neighbor search between the following consecutive DAIs. **(A)** Random selection of cluster centers with at least distance dmin from each other. **(B)** Cluster labeling with the nearest neighbor search. **(C)** Label removal from clusters with less than N points (*N* = 6 in this example). **(D)** Relabeling of unlabeled points. **(E)** Calculate the cluster median coordinates with respect of all axis (♢). End of the initial clustering (t_i_). **(F)** Next data acquisition (t_i+1_) with new points marked as gray. **(G)** Labeling of new (t_i+1_) points based on their nearest neighbor label. **(H)** Calculation of cluster median locations in t_i+1_ (♢). **(I)** Calculation of cluster median location differences between t_i+1_ (opaque ♢) and t_i_ (transparent ♢).

The working principles of the initial clustering and cluster center monitoring are presented with a synthetic example in Figure 3. The initial clustering phase was conducted in two steps (Figures 3A–D). First, Dart Throwing Poisson Disk sampling as presented by Chambers (2013) was used. Euclidean clustering was then applied to the sampled point cloud. Only 3D coordinate information was used as an input in clustering processes. First, a spatial kd tree (with *k* = 3) was built for the point cloud. All the neighbors within the preset clustering distance were then listed for every point. All points were then randomly listed. Each point was then selected individually, starting with the new listing order. If none of the selected points' neighboring points had been chosen before, it was classified as a cluster seed point with an increasing label number. When all the list's points had been passed, a nearest neighbor search was performed for the object point cloud, and all non-classified points were labeled according to their nearest cluster seed point. To make clustering more robust, labels for points assigned to small clusters were removed. The nearest neighbor search was then performed again, using all labeled points. Here, the initial cluster diameter was set at 0.15 m, and the smallest cluster size allowed in initial clustering was set at 100 points.

After clustering the first DAI, the points of those following it were loaded, pre-processed, and clustered using its nearest neighbor labels. Clustering continued as with the previous DAI, storing the cluster center locations for temporal analysis (Figures 3F–H). In total, 41 DAIs were included in the analysis. The cluster center points were defined here as the median value of all points in a point cluster calculated separately for each coordinate axis (Figure 3E). Median coordinate values were used to limit the effect of possible outlier points in clusters. The cluster median coordinates of each DAI could then be compared with the coordinate values of either the initial or previous DAI. The former option determines a cluster's total displacement from its initial position, while the latter reveals the movement amplitude and directionality between DAIs. Here, the former comparison was undertaken in results analysis (Figure 3I).

## Results

### Measurement Stability

Object stability between scans was verified by comparing the point number and the location of the five static reference spheres against their initial values. The stability testing results showed that the point number standard deviation between scans was <1% for all reference spheres, and their center displacement was on average up to 1.2 mm in all spheres. The main indication in the point cloud stability test was that during measurements laser scanners performed consistently within their reported precision limits, regardless of their different wavelengths. Detailed results for measurement stability testing are given in Supplementary Datasheet 1.

### Cluster Movement Analysis of the *Large* Norway Maple Point Clouds

**Figure 4:**
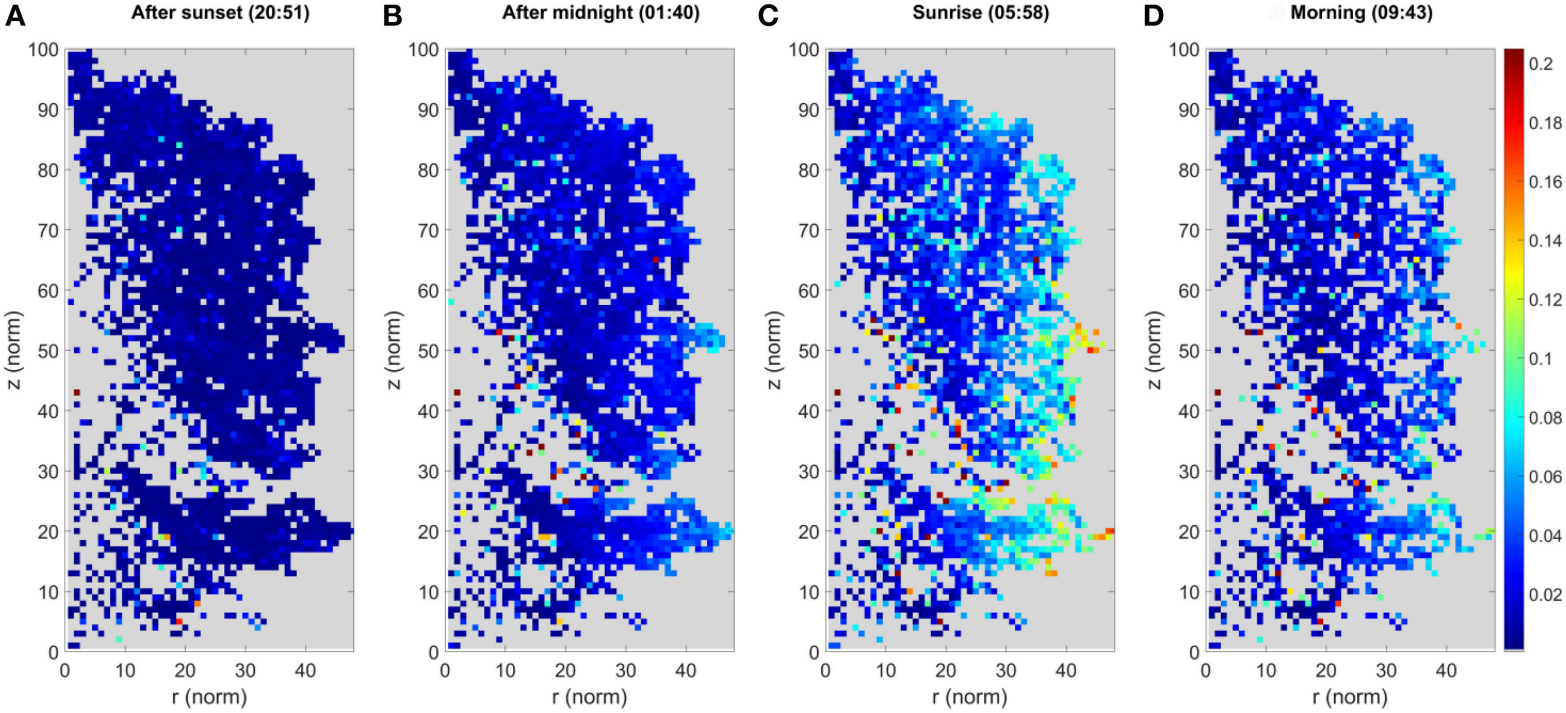
The maximum cluster displacement of the *large* Norway maple. Each image panel **(A–D)** represents the aggregated point cloud of the ***large***Norway maple (*Acer platanoides*) projected onto normalized cylindrical coordinates. The colors depict the maximum cluster displacement for the cluster location after the first DAI at 20:10 h. The color of each pixel shows the maximum displacement of all cluster centers located within it in meters. Coordinates are presented as the normalized cylindrical coordinates, as defined in the text.

Cluster movements were measured as Euclidean 3D distances. They were analyzed by monitoring how each cluster center point moved over the DAIs with respect to the initial cluster center in the first DAI. Cluster movement results for the *large* Norway maple are illustrated in Figure 4. The illustration was created as follows: (i) projecting all cluster centers a in cylindrical coordinate system (r, ϕ, z) centered on a manually selected vertical principal axis following the stem of the studied tree; (ii) dividing the target into a height-normalized grid in a (r, z) plane, where [(r_norm_, z_norm_) = (r, z)^*^100/z_max_]; (iii) labeling all cluster centers in a grid cell; and (iv) presenting the aggregated result in the normalized (r_norm_, z_norm_) plane, with the color of each pixel being the maximum displacement from its initial cluster center.

The results for the *large* Norway maple show that the maximum cluster movements detected are mostly within a few centimeters off their initial locations around sunset (20:51 h.). After midnight (01:40 h.) the entire maple foliage shows systematic cluster movements pronounced toward the crown edges, where larger branch tips have moved up to 0.08 m. The maximum displacements are detected around sunrise (05:58 h.), where the outermost clusters have been displaced by at least 0.20 m, and outer areas of the foliage show systematic movements between 0.08 and 0.16 m. The inner foliage closer or next to the maple stem demonstrate <0.05 m displacement. After sunrise (09:43 h.) the branches and leaves return to their initial, pre-sunset positions. The inner foliage is displaced by of 0.05 m or less. The outer tips of branches still present maximum displacement of up to 0.14 m.

As the *large* Norway maple has dense foliage, the cluster number is lower inside the crown because of internal occlusions. This is shown as an empty space in Figure 3. Moreover, the figure shows randomly located individual pixels within the tree crown, with clearly higher displacement values than those of their neighboring pixels. These results also come from internal occlusions: Individual clusters within the tree crown may lose points due to leaf and branch movement between the DAIs, yet collect new stray points. Previously occluded branch, leaf, and stem parts may also be included in clusters.

**Figure 5:**
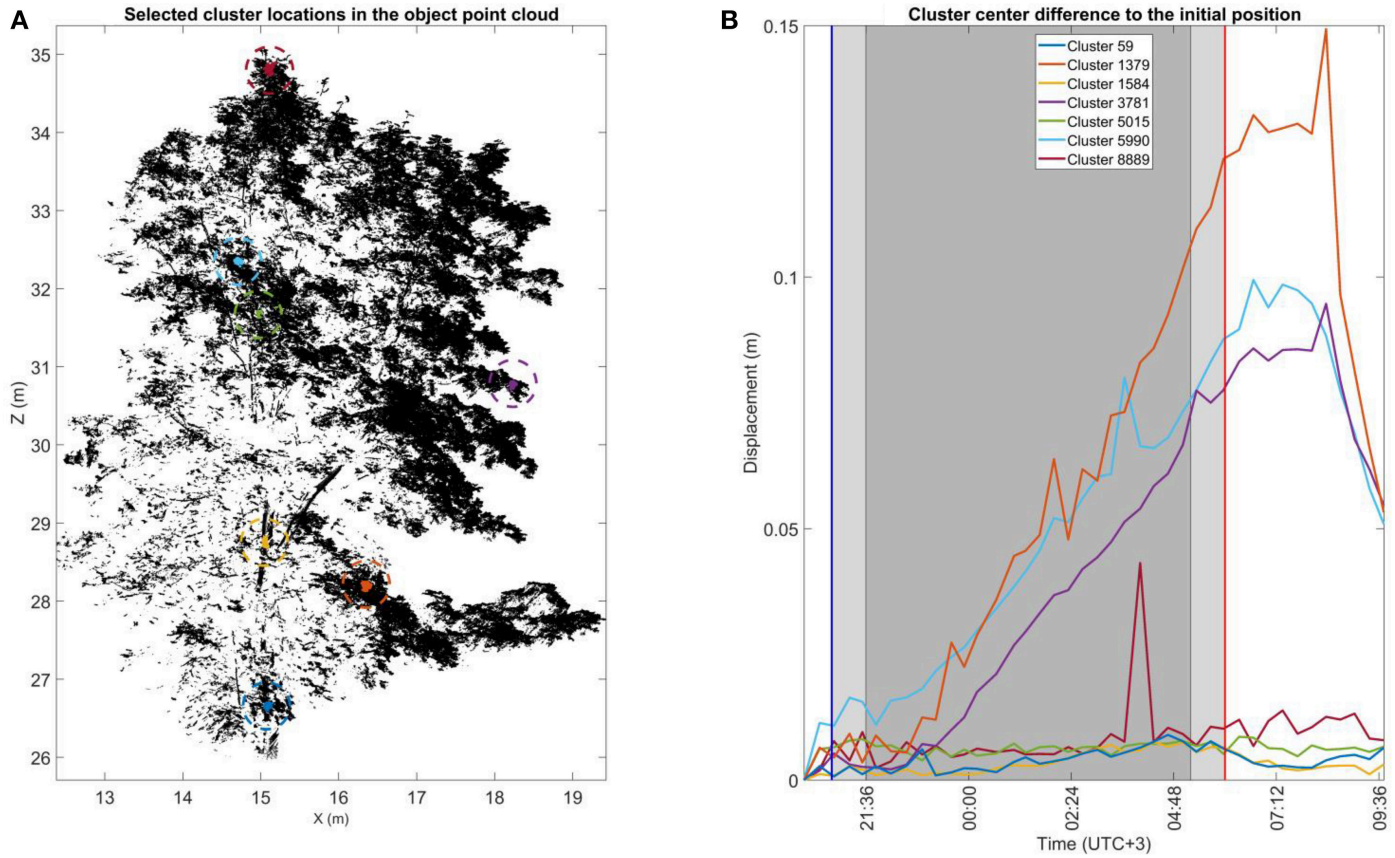
The cluster displacement over time of selected clusters in the ***large***Norway maple. **(A)** The selected cluster location in the ***large***Norway maple point cloud during the initial scan during the first DAI at 20:10 h. Sizes of the selected cluster points have been highlighted for visual clarity. **(B)** Cluster center displacement from their initial location measured at 20:10 h. The blue and red vertical lines mark the times of sunset (20:48 h.) and sunrise (06:00 h.). The light shaded area after sunset and before sunrise shows civil twilight. The dark shaded area shows the time of nautical and astronomical twilights when the measurement scene was visually dark.

To demonstrate cluster displacements over time at the individual cluster level, Figure 5 illustrates the total 3D displacements of seven manually selected clusters in the *large* Norway maple. The *large* Norway maple was segmented in a total of 8,911 clusters. Three of the clusters were selected from different parts of the *large* Norway maple stem [59 (blue), 1,584 (yellow), and 5,015 (green)]. The four other clusters [(1,379 (orange), 3,781 (lilac), 5,990 (cyan), and 8,889 (dark red)] were selected from the different parts of the maple crown's outer edge. Displacement patterns for all cluster components of all three targets are available in Supplementary Datasheet 2.

All stem clusters display a similar displacements trend. Their maximum displacements remained around 0.01 m over the whole monitoring period. They thus demonstrated the expected stem stability.

Three of the four crown clusters illustrate similar displacement patterns, with some variation. Displacement from the initial DAI position is within 0.01 cm until about 23:00 h. The displacement amplitudes then start to increase in a relatively linear trend until reaching their maximums after sunrise. The respective maximum displacement amplitudes are 0.09 m (lilac and cyan) and 0.15 m (orange). When the maximum displacements had been reached, all three crown clusters began to rapidly return toward their initial position, which was not completely captured within the monitoring period.

The fourth selected crown cluster (dark red) shows a displacement trend deviating from the other crown clusters. This more resembles those of stem clusters. Its displacement value remains around 0.01 m over the whole monitoring period, excluding a single DAI around 03:00 h. At this DAI, the cluster displacement value increases temporarily to 0.04 m but returns to the trend in the following DAI. This suggests that the recorded displacement value results from a temporary disturbance such as local airflow. Another crown cluster (5,990, cyan) deviates similarly in its trend at the same DAI, but this does not stand out significantly. Location is the main difference between the fourth crown and other crown clusters. This cluster was at the top of the *large* Norway maple crown, and it was closest to the crown center in the horizontal (xy). It was therefore closer to the stem than the other crown clusters.

### Cluster Movement Analysis of the *Small* Norway Maple Point Clouds

**Figure 6:**
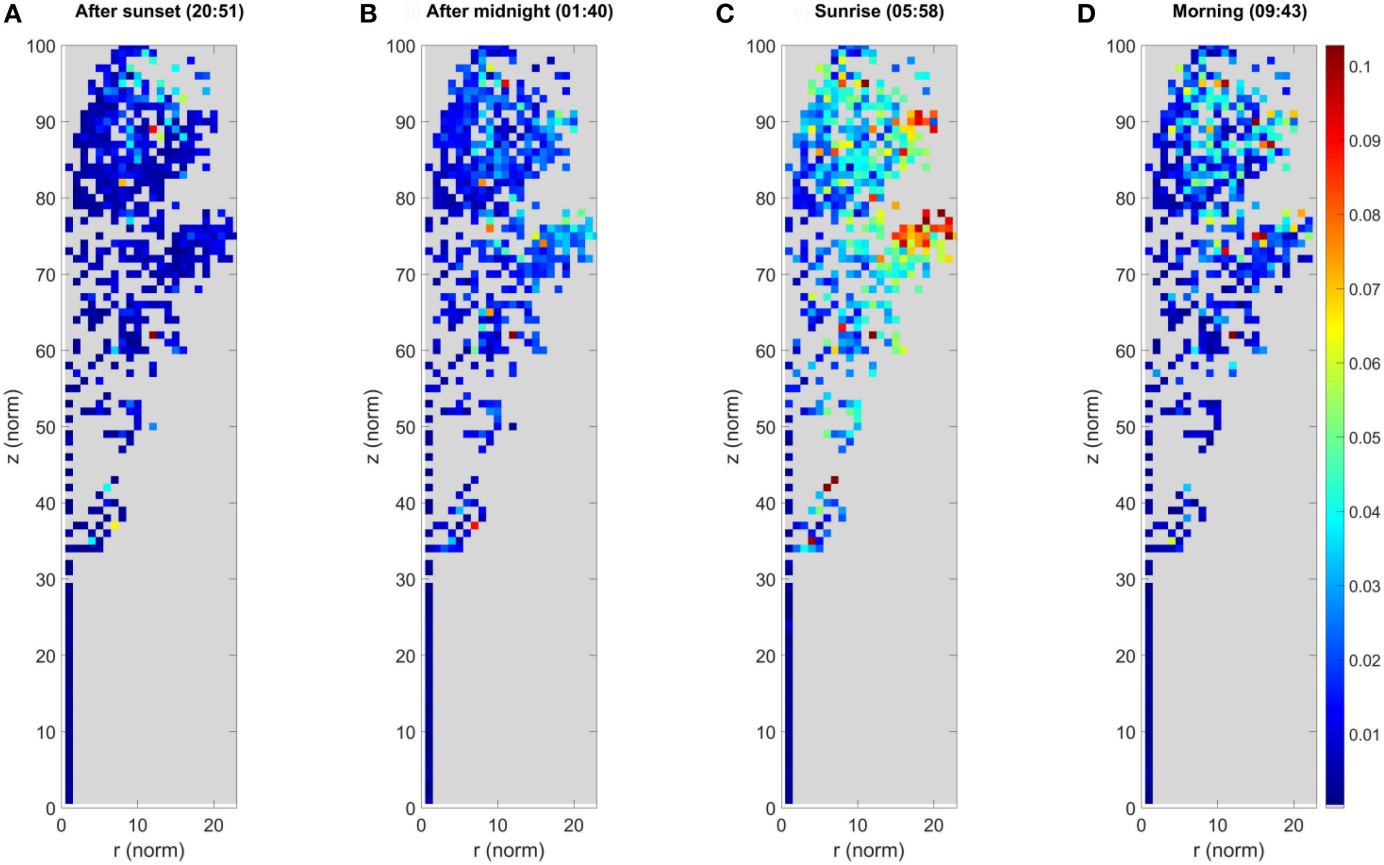
The maximum cluster displacement of the ***small***Norway maple. Each image panel **(A–D)** represents the aggregated point cloud of the ***small***Norway maple (*Acer platanoides*) projected onto normalized cylindrical coordinates. The colors depict the maximum cluster displacement for the cluster location after the first DAI at 20:10 h. The color of each pixel shows the maximum displacement of all cluster centers located within it in meters. Coordinates are presented as the normalized cylindrical coordinates, as defined in the text.

The *small* maple point clouds were segmented into a total of 877 individual clusters. Cluster movement results for the *small* Norway maple are illustrated in Figure 6, following the same procedure as in Figure 4. The results for the *small* Norway maple show that the detected maximum cluster movement pattern is similar to that detected in the *large* Norway maple point clouds. The maximum cluster movements are largely confined to within a few centimeters of their initial locations around sunset (20:51 h.). After midnight (01:40 h.), the *small* maple foliage shows systematic cluster movements pronounced toward the crown edges. The largest cluster displacements from their initial positions are about 0.06 m when the outlying clusters are excluded. As with the *large* Norway maple, absolute maximum cluster displacements are detected around sunrise (05:58 h.), when the outermost crown clusters show displacements of at least 0.09 m. The remaining foliage shows systematic movements of between 0.05 and 0.09 m. Clusters in the inner foliage and on the stem show displacements of <0.03 m. After sunrise (09:43 h.) the branches and leaves are return to their daytime positions. Displacements in the inner foliage are within 0.03 m or less. Crown edge clusters on top of the *small* Norway maple still systematically show maximum displacements of 0.06 m from their initial evening positions.

**Figure 7:**
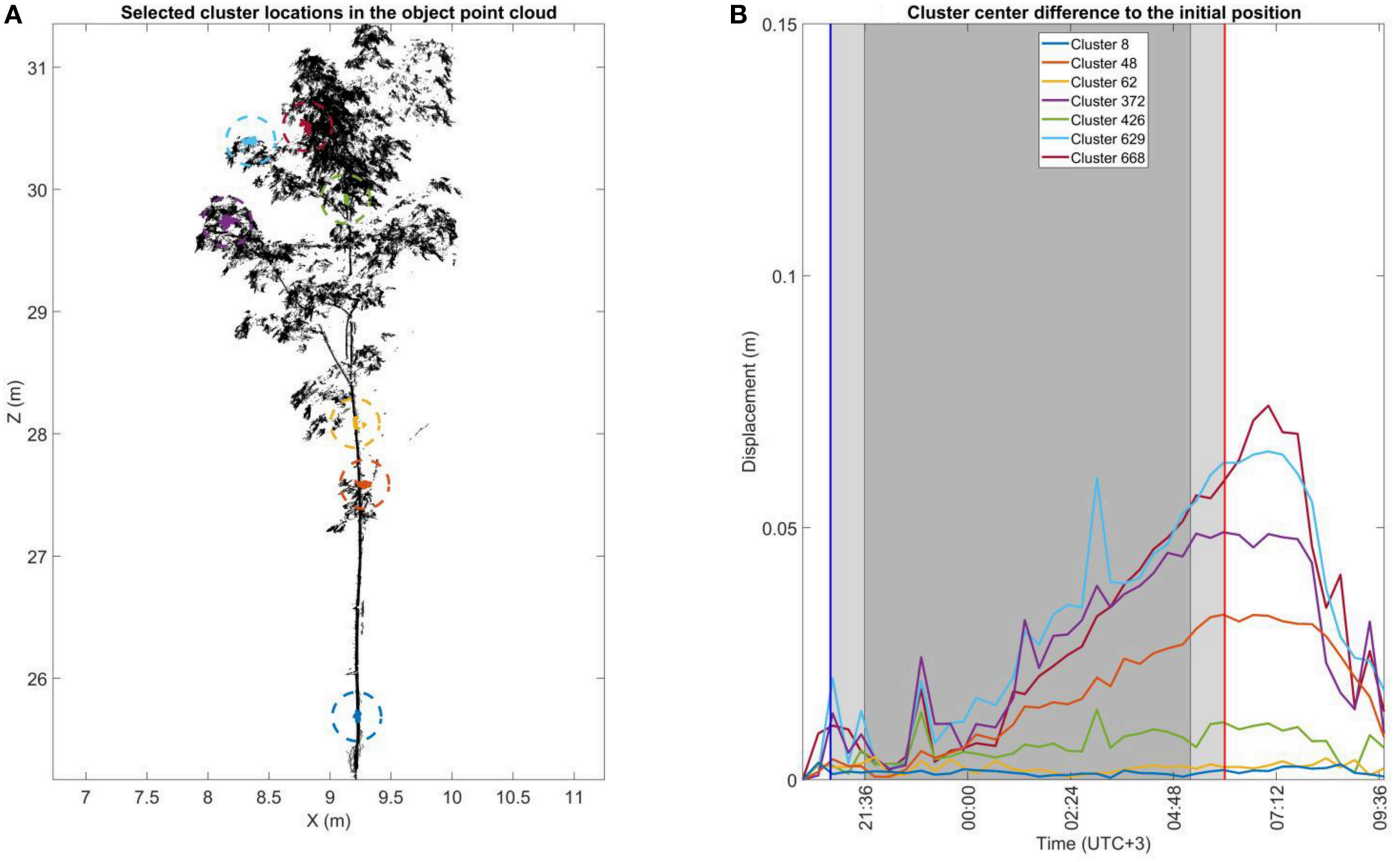
The cluster displacement over time of selected clusters in the ***small***Norway maple. **(A)** The selected cluster location in the ***small***Norway maple point cloud during the initial scan during the first DAI at 20:10 h. Sizes of the selected cluster points have been highlighted with a dashed circle for visual clarity. **(B)** Cluster center displacement from their initial location measured at 20:10 h. The blue and red vertical line mark the times of sunset (20:48 h.) and sunrise (06:00 h.). The light shaded area after sunset and before sunrise shows civil twilight. The dark shaded area shows the time of nautical and astronomical twilights when the measurement scene was visually dark.

Like Figures 5, 7 illustrates the total 3D displacements of seven manually selected clusters in the *small* Norway maple. The *small* Norway maple was segmented in a total of 877 clusters. Here, three stem clusters (8, 62, and 426) and four crown clusters (48, 372, 629, and 668) were selected. The two lower stem clusters (8 and 62) show basically no displacement within measurement limits over time. The highest stem cluster (426), located in the upper part of the maple crown, shows time-dependent displacement limited to a maximum of around 0.01 m.

All four crown clusters illustrate a similar displacement patterns. Until around midnight (00:00 h.) cluster displacements are within 0.01 m. Displacement values then begin to increase until they reach their maximums about an hour after sunrise. Cluster displacement values then fall rapidly toward the initial, pre-sunset DAI positions. The location of the cluster in the crown clearly affects the displacement amplitude. The lowest cluster close to the stem (48, orange) shows the most limited maximum displacement at about 0.03 m. The second lowest cluster (372, lilac) shows a maximum displacement of about 0.05 m. The displacements of the two uppermost crown clusters (629 and 668, cyan and dark red) extend to a maximum of 0.08 m.

The cluster displacement trend is similar for all clusters but presents occasional spiking at individual DAIs. Around 23:00 h., all clusters in the upper part of the *small* Norway maple were similarly affected. This suggests a possible airflow affecting the whole *small* Norway maple crown during the scan. The detected spiking effects are nevertheless limited to individual DAIs and cause deviations of only 0.01–0.02 m from the general displacement trend.

### Cluster Movement Analysis of the Lamppost Point Clouds

Corresponding cluster movement results for the reference target, the lamp post, are reported in Supplementary Datasheet 1. The lamppost was segmented in 124 individual point cloud clusters using the same parametrization as the two Norway maple targets. Lamppost results were within the expected measurement limits. A clear majority of all detected maximum cluster movements were 6 mm or less at all DAIs. The lamppost results verify that individual and overall cluster displacement findings in the target Norway maple crowns are the result of their internal structural dynamics, not of point cloud or clustering instability.

## Discussion

### Comparison of Cluster Movement Between the Two Norway Maples

A comparison of the cluster displacement trends in the two Norway maples showed both similarities and differences. In both maples stem clusters were virtually stable within measurement accuracy over the duration of the measurement. Crown clusters also showed similar displacement trends over time.

The main difference between the Norway maples was that the detected cluster movement amplitudes were more pronounced for the *large* Norway maple (note that the scale for the y-axes is the same in both figures). Additionally, DAI-long deviations from the general trend in the selected clusters were target-specific and did not occur in the same DAIs in the Norway maples. This suggests localized airflows—the maples were located within 10 m of each other—clustering-related anomalies, or internal dynamics specific to an individual specimen as Zlinszky et al. (2017) suggest.

In summary, the experiment demonstrates that different specimens of the same tree species evince both shared and differentiating temporal patterns in their structural dynamics under the same measurement conditions. The drivers of individual variation and their effect on the dynamics amplitude are important for species-specific modeling but require more systematic measurements beyond the scope of this study, which focuses on methodology.

### The Proposed Workflow Efficiency With Regard to Previous Research

Recent studies monitoring circadian rhythms in vegetation using TLS time series have raised the need for techniques which effectively monitor phenomena outside laboratory conditions (Puttonen et al., 2015, 2016; Zlinszky et al., 2017). Capturing and locating these systematic temporal plant movements require high-density TLS data. These are collected at hourly or even sub-hourly intervals with centimeter spatial resolution—and even less for targets within a few dozen meters. Fulfilling these requirements rapidly becomes data-intensive when fully grown plants are used, and the study area is extended to standard ecological study units like forest plots where detection distances increase to dozens meters.

Promising results have been presented for monitoring diurnal characteristics of small potted plants in controlled environments with close-range laser scanning (e.g., Dornbusch et al., 2012, 2014). Dornbusch et al. observed leaf hyponasty in an *Arabidopsis thaliana* specimen in their studies, with a special phenotyping system over several days. One of their findings showed a rapid downward movement of leaves shortly after dawn. Wiese et al. (2007) have also previously reported this. They interpreted this movement as correlated with increased leaf growth. The displacement maximum of point cloud clusters in Norway maples was also detected after sunrise in this study. However, measurements were taken at the end of the growth season about 1 month before abscissions, suggesting another mechanism behind the movement.

Herrero-Huerta et al. (2018) studied the leaf movements of two potted *Calathea roseopicta* plants in both natural lighting and in darkness. They found that leaf-movement patterns were attenuated and different in darkness than in natural lighting conditions. They used an octree-based segmentation method to monitor movements of each plant leaf from within the point cloud and point cloud convex hulls to determine volumetric change in the point clouds. These approaches are suitable for their selected measurement setting. There were high local point densities and a plant species with few large leaves that could be clearly distinguished at all times even with a single TLS system. In this study the measurement setting was more complex: The measurement scene had more targets, more internal and external occlusions, longer distances between targets and scanners, and a greater variation in point densities. A more straightforward clustering framework was therefore selected. The convex hull approach determining volumetric changes is as also less beneficial for larger plants. Although the leaves and branches of fully grown trees show clear circadian rhythm dynamics of up to dozens of centimeters, the relative volumetric change compared with the whole crown volume is limited. In general, considering the technical strengths and limitations of TLS mentioned in the introduction, the TLS and image-based techniques can be considered complementary: Imaging is near instantaneous compared with point cloud collection, and imaging sensors can usually detect a set of wavelength channels ranging from a few channels to hundreds. However, imaging sensors require external light. A detailed 3D model from images based on terrestrial close-range photogrammetry is possible (e.g., Li et al., 2013; Lou et al., 2014; Herrero-Huerta et al., 2015; Nevalainen et al., 2016). However, it requires several images of the object with high image overlap, complicating image collection especially over multiple DAIs. Drones enable time-efficient image collection and can provide photogrammetric point clouds of even 15,000 points/m^2^ when there is a low flying altitude, and a high-resolution camera is used (Viljanen et al., 2018). However, aerial drone imaging has limitations in reconstructing 3D models under branches and within the crown because there is no of visual line of sight. Mikita et al. (2016) suggested using a combination of drones and terrestrial imaging as a solution for creating complete 3D models. However, point cloud registration in dense canopies can be problematic because of reference point occlusion. These limitations make imaging approaches difficult to apply to large plants in outdoor conditions.

The results demonstrate that the presented workflow can be used to monitor point cloud cluster movements consistently on object surfaces when the line of sight to an individual cluster is retained throughout monitoring. Line of sight to individual clusters was lost due to internal occlusion dynamics in the measurement scene (e.g., systematic branch movements in the trees).

Occlusion effects can be reduced with suitable cluster size selection, but the cluster size also affects movement detection sensitivity. Other ways to mitigate occlusions are capturing denser point clouds and increasing the scan location number to obtain better coverage of the studied targets. The former leads to longer acquisition times; the latter quickly becomes impractical due to resource and acquisition time limitations. In this study occlusion did not significantly affect the overall aggregated results.

The clustering approach tested in this study arguably has an advantage over voxelization approaches for monitoring internal dynamics in plant point clouds over a diurnal cycle. For example, Lecigne et al. (2017) presented a voxelization-based method to analyze tree crown arrangements and their architectural traits. The voxelization approach is more straightforward and faster than the randomized range-search-based clustering used in this study, but clustering is only performed in the initial DAI. Clustering in the following DAIs is then undertaken with a simple nearest neighbor search, which is faster to perform. This makes cluster presentation responsive to the intrinsic plant dynamics, because unlike voxels cluster locations and sizes are not fixed in space. However, the data acquisitions need to be planned with short enough intervals to make sure that cluster movement amplitudes between two DAIs do not lead to overlapping clusters.

The presented method improves the monitoring performance compared with the earlier studies of Puttonen et al. (2016) and Zlinszky et al. (2017), which focus on short interval TLS point cloud uses. In both studies circadian rhythms in tree crowns were monitored for changes in point cloud height percentiles. Height percentile monitoring offers a statistical interpretation of crown movements. This may be the only feasible option with sparse point clouds, but it cannot distinguish varying movement patterns in different parts of the crown. As this study shows, clusters in horizontal branch tips present the highest movement amplitudes. Clusters on the same horizontal branch but closer to the stem move less. In height percentile presentation these effects would be averaged and decrease the total movement amplitude. However, clusters near vertically aligned branch tips may present very limited movement, even though their distance to the stem may be similar to those on horizontal branch tips.

This study's results also raise a question concerning the optimal timing for large-area data acquisition, e.g., in forest inventory. As the results show, the highest detected movement amplitudes occur *after* sunrise, when the measurement scene is already well lit. This may have implications for the planning of several day-long measurement campaigns over wide areas, because crown geometries will change during data acquisition, adding noise to the dataset and bias depending on the forest type. Multi-temporal studies focusing on quantifying canopy changes in monthly or annual intervals are another area which would benefit from a consideration of the study's results. To our knowledge few if any studies in the literature have accounted for the possibility of circadian rhythm dynamics in tree crowns. For example, Martin-Ducup et al. (2017) studied tree response to surrounding gaps in a bi-temporal study with a 2-year interval. One of their study targets was a sugar maple (*Acer saccharum* Marsh.) presenting more than 0.40 m branch movements in horizontal branch tips over the acquisition period. Here, point cloud clusters on a similar sized Norway maple presented movements of up to 0.17 m (99th percentile) over a 9-h period. Knowledge of the different structural dynamic mechanisms in tree crowns will thus be very important for differentiating between short- and long-term components.

Overall, monitoring structural plant dynamics in the natural environment is a technically complex issue due to several intrinsic and external variables. Individual- and species-related dynamics, limited data capture time, large data quantities, and weather conditions all have effects that need consideration in developing future spatio-temporal models.

## Conclusions

Terrestrial laser scanning time series represent a new approach for the study of circadian rhythm dynamics in plant sciences and are increasingly utilized in experimental settings. An ability to capture and monitor individual plant-part dynamics in a systematic and highly automatized manner will be essential for developing new dynamic structural models able to cover whole plants in their natural environment. It is therefore highly important that the large datasets collected during laser scanning time series experiments can be exploited to connect the new information about plant dynamics with the already existing knowledge of circadian rhythm characteristics. We have presented a data collection framework for monitoring the circadian rhythm in spatio-temporal dynamics of tree crowns in the growing environment to support future research on the topic.

## Author Contributions

EP and NP contributed to the study's conception and design. EP conducted data analysis and method development, and wrote the manuscript's first draft. EP, ML, PL, RN, ZF, XL, SW, MP, TH, and MK contributed to the data acquisition campaign. All the authors contributed to manuscript revision, read, and approved the submitted version.

### Conflict of Interest Statement

The authors declare that the research was conducted in the absence of any commercial or financial relationships that could be construed as a potential conflict of interest.

## Supplementary Material

Supplementary Video 1 A time-lapse video of measurements, 20:10–07:00 h. The time lapse was recorded next to the FARO Focus 3D 120 S1 (marked with blue in Figure 1).

Supplementary Datasheet 1 Measurement stability results and cluster displacement analysis of the reference target, the lamppost.

Supplementary Datasheet 2 Cluster movement tables for all presented objects in ASCII format, zip compressed (.txt). **Scan times.txt** contains scan DAIs in format “DD-MMM(txt)-YYYY hh:mm:ss”, with one DAI in each line. **Ref_sphere_ii_center.txt** files contain the center point locations fitted to the reference sphere point clouds. Each sphere has its own file, labeled “ii” in the file name. The file format is that each row corresponds with the DAIs, and the column order of the coordinates is x, y, and z. For all three monitored targets, there are three files named “**TT_cluster_centers_x**.**txt**,” where “TT” is the target name and the cluster coordinate axis is at the end. The file format for the target cluster centers is as follows: Row numbers label the ID of corresponding individual cluster centers. Each column corresponds with the DAIs listed in **Scan_times.txt** in the same order. If a cluster center has the value “NaN,” then that cluster has been occluded over time. All coordinate values are reported in the georeferenced coordinate system.
