# Supplementary material for: A Clustering Framework for Monitoring Circadian Rhythm in Structural Dynamics in Plants From Terrestrial Laser Scanning Time Series
Source: Front Plant Sci. 2019 Apr 17;10:486. doi: 10.3389/fpls.2019.00486 (PMC6499199; doi:10.3389/fpls.2019.00486)
Supplement: Supplementary Datasheet 1 — Measurement stability results and cluster displacement analysis of the reference target, the lamppost. [file Data_Sheet_1.pdf]

Supplementary Material

A Clustering Framework for Monitoring Circadian Rhythm in Structural Dynamics in Plants from Terrestrial Laser Scanning Time Series

Eetu Puttonen[1,2], Matti Lehtomäki[1], Paula Litkey[1], Roope Näsi[1], Ziyi Feng[1], Xinlian Liang[1], Samantha Wittke[1,3], Miloš Pandžić[4], Teemu Hakala[1,2], Mika Karjalainen[1,2] and Norbert Pfeifer[5]

[1]Finnish Geospatial Research Institute, Department of Remote Sensing and Photogrammetry, National Land Survey of Finland, Helsinki, Finland

[2] Centre of Excellence in Laser Scanning Research, Department of Remote Sensing and Photogrammetry, National Land Survey of Finland, Helsinki, Finland

[3]Department of Built Environment, Aalto University, Espoo, Finland

[4] University of Novi Sad, BioSense Institute, Novi Sad, Serbia

[5]Department of Geodesy and Geoinformation, Technische Universität Wien, Vienna, Austria

*** Correspondence:** Corresponding Author: eetu.puttonen@nls.fi

1 Supplementary Data

1.1 Measurement stability results

Object stability between scans was verified by comparing the point number and the location of the five static reference spheres with their initial values. **Supplementary Figure 2-1** shows the variances of the reference spheres' relative point number and the location of their centers. The object point numbers correspond with those remaining after point cloud merging and before clustering steps. Reference sphere centers were calculated using Alan Jennings's sphere fitting method (available at www.mathworks.com/matlabcentral/fileexchange/34129). Stability testing results show that the point number standard deviation between scans was less than 1% for all reference spheres and that their center displacement was an average of to 1.2 mm for reference sphere 3 and less than 1 mm for all other spheres. For the five monitored objects point cloud size variations were up to 2.3% over the experiment period. This can be considered reasonable given the object sizes and their structural complexity. These cloud size variations represent a clear improvement on the values reported in an earlier study (Puttonen *et al.*, 2016), where corresponding point cloud size variations were 6.5% and 7.7% at two different measurement sites.

The main indication of the point cloud stability test was that the laser scanners performed consistently during measurements, and that their performance was within their reported precision limits, regardless of their different wavelengths. It can therefore be assumed that the data collection

procedure did not significantly affect point clouds, which would have led to additional cluster movements over the course of the experiment.

1.2 Maximum cluster displacement map of the reference target, the lamp post

For reference the corresponding cluster movement results for the lamp post are illustrated in **Supplementary Figure 2-2**. As expected, the lamppost results show that the clear majority of all detected maximum cluster movements are 6 mm or less at all depicted measurement times. Some pixels show displacements of over 1 cm, but these are located on the outer edge of the lamppost pillar and are likely rasterization artifacts, where few points vary between the scans. The top of the lamppost shows a modest increase in displacement values toward sunrise and morning. This may result from increased variance in point cloud due to surface condensation, i.e. water droplets adding to surface roughness. However, all the variability increases are of only a few millimeters which is still within general measurement limits. Thus, the lamppost can be considered a stable object, showing that movements detected in the Norway maple point clouds are to the result of dynamics inside the tree foliage.

The individual cluster movement results for the lamppost are illustrated in **Supplementary Figure 2-3**. As with the two Norway maple targets, seven clusters were selected from the lamppost point clouds, and their displacement from the initial DAI were monitored. For all clusters the maximum displacement over time was less than 0.01 m. This confirms that the clustering results are stable, and the clustering method itself causes no systematic drift in the absence of internal occlusions between consecutive DAIs.

2 Supplementary Figures

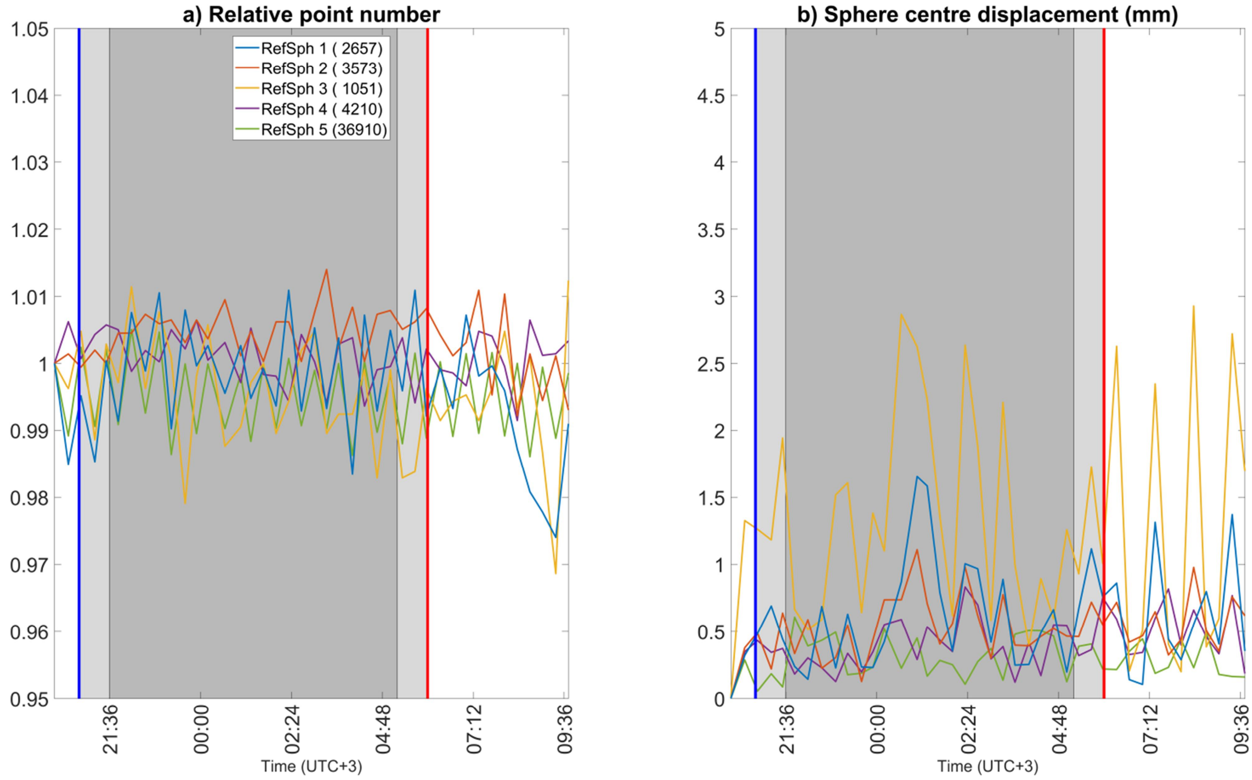

Supplementary Figure 2-1. The figure shows **a)** the relative point number variation in reference sphere point clouds and **b)** the displacement of the sphere centers fitted in their point clouds over time using the initial scan (20:10 h.) as the reference. The blue and red vertical lines mark the times of sunset (20:48 h.) and sunrise (06:00 h.). The bracketed number after the sphere number in **a)** shows the combined point count of the initial DAI. The light shaded area after sunset and before sunrise shows civil twilight. The dark shaded area shows the time of nautical and astronomical twilights when the measurement scene was visually dark.

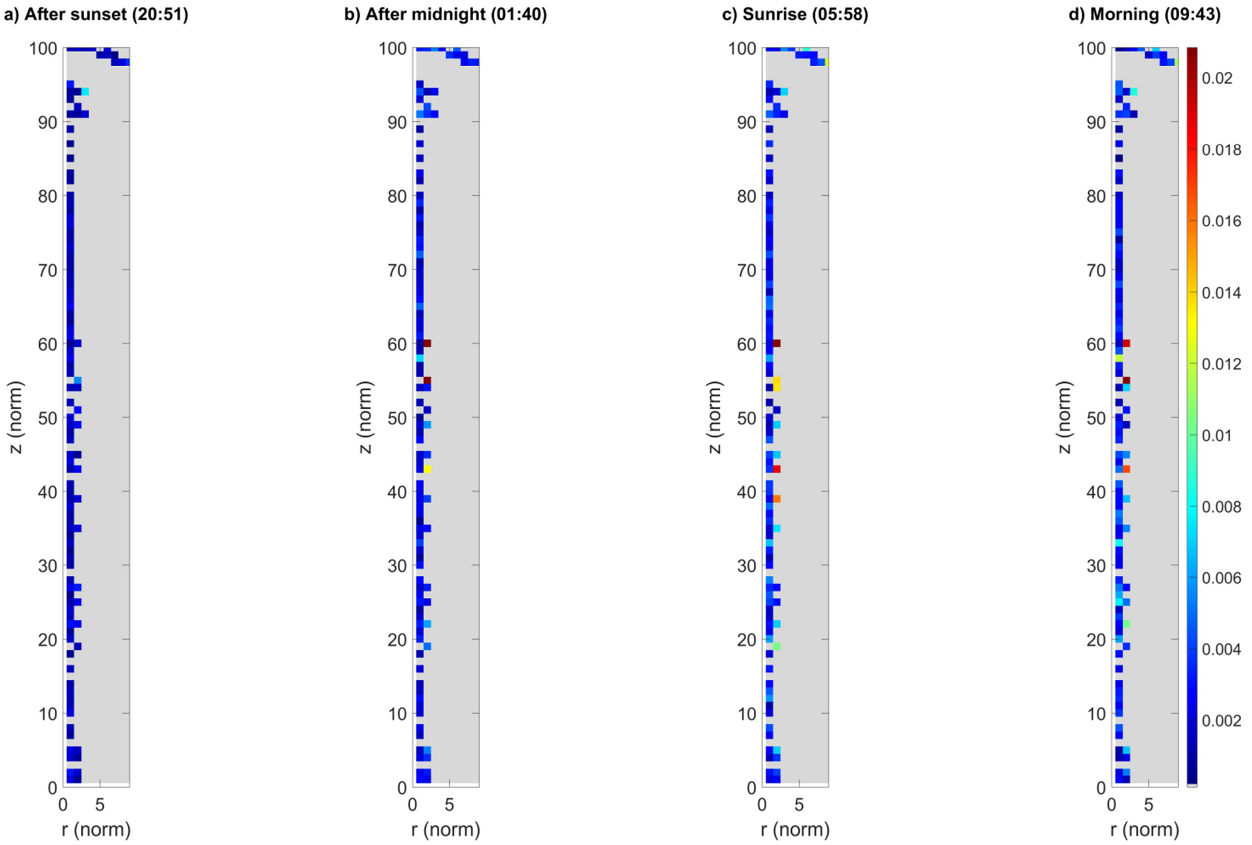

Supplementary Figure 2-2. The maximum cluster displacement in the lamppost point cloud (**a** – **d**). The images represent aggregated the point cloud of the lamppost projected onto normalized cylindrical coordinates. The colors depict the maximum cluster displacement for the cluster location after the first DAI at 20:10 h. Each pixel shows the maximum displacement of all cluster centers located within it. Color scale values are in meters. Normalized cylindrical coordinates are defined in the text.

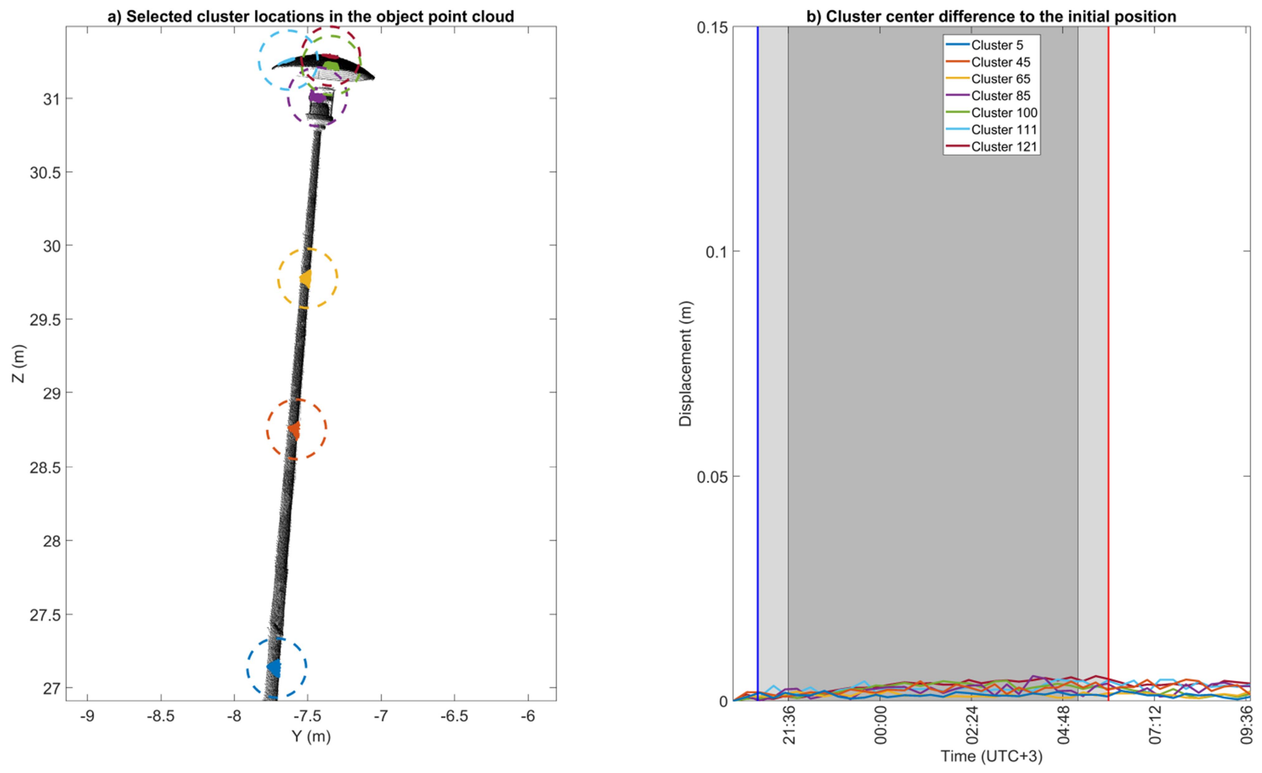

Supplementary Figure 2-3. The cluster displacement over time of selected clusters in the reference target, the lamp-post. **a)** The selected cluster locations in the lamppost point cloud during the initial scan during the first DAI at 20:10 h. Sizes of the selected cluster points have been highlighted and increased for visual clarity. **b)** Cluster center displacement from the initial location, measured at 20:10 h. The blue and red vertical lines mark the times of sunset (20:48 h.) and sunrise (06:00 h.). The light shaded area after sunset and before sunrise shows civil twilight. The dark shaded area shows the time of nautical and astronomical twilights when the measurement scene was visually dark.
